# Supplementary material for: Transcriptome change of Staphylococcus aureus in infected mouse liver
Source: Commun Biol. 2022 Jul 20;5:721. doi: 10.1038/s42003-022-03674-5 (PMC9300722; doi:10.1038/s42003-022-03674-5)
Supplement: Supplementary file 2 — Supplementary information [file 42003_2022_3674_MOESM2_ESM.pdf]

Supplementary information

Transcriptome change of *Staphylococcus aureus* in infected mouse liver

Hiroshi Hamamoto[1,#], Suresh Panthee[2,3#], Atmika Paudel[4], Suguru Ohgi[5,6], Yutaka Suzuki[7], Koichi Makimura[1], Kazuhisa Sekimizu[2,*]

[1] Teikyo University Institute of Medical Mycology, 359 Otsuka, Hachio-ji shi, Tokyo, 192-0395, Japan

[2] Drug Discoveries by Silkworm Models, Faculty of Pharma-Science, Teikyo University, 359 Otsuka, Hachio-ji shi, Tokyo, 192-0395, Japan

[3] Current address: GenEndeavor LLC, 26219 Eden Landing Rd, Hayward, CA, 94545

[4] International Institute for Zoonosis Control, Hokkaido University, North 20, West 10, Kita-ku, Sapporo Hokkaido 001-0020, Japan

[5] Laboratory of Microbiology, Graduate School of Pharmaceutical Sciences, The University of Tokyo, 7-3-1 Hongo, Bunkyo-ku, Tokyo 111-0033, Japan

[6] Current address: Kyowa Kirin Co., Ltd., 1-9-2 Otemachi, Chiyoda-ku, Tokyo 100-0004, Japan

[7] Department of Computational Biology and Medical Sciences, Graduate School of Frontier Sciences, The University of Tokyo, 5-1-5 Kashiwanoha, Kashiwa shi, Chiba, 277-8562, Japan

Corresponding Author:

* To whom correspondence should be addressed (sekimizu@main.teikyo-u.ac.jp)

[#] These authors equally contributed to this study

Sample Origin	in liver									*in vitro*			
Harvested time/Medium	6 h.p.i.			24 h.p.i			48 h.p.i.			TSB			
Sample name	No.3	No.23	No.33	No.3	No.13	No.83	L10-2	L10-3	D2-3	NMWT-1	NMWT-2	MMWT-3	NMWT-4
cells in the sample ($x10^3$)	1,484	1,300	1,162	503	143	753	3,580	5,001	3,561				
Total reads	178,491,141	98,324,305	179,892,351	105,370,467	88,463,592	106,305,171	104,816,965	93,625,792	89,721,859	19,256,012	19,700,338	25,971,088	44,444,034
Mapped reads	328,107	526,721	237,715	534,170	404,538	576,442	980,823	1,149,746	249,904	16,519,511	16,578,709	22,522,546	33,925,903
Uniquely mapped reads	300,156	485,379	215,196	423,322	315,772	489,315	707,532	533,073	161,085	587,376	5,596,383	7,368,820	4,030,565
Non-specifically mapped reads	27,951	41,342	22,519	110,848	88,766	87,127	273,291	616,673	88,819	10,629,133	10,982,326	15,153,726	29,895,338
The genes not read mapped	374 (12%)			269 (9.0%)			14 (0.47%)			97 (3.2%)			

Supplementary Table 1| Summary of *in vivo* RNA-Seq results of *S. aureus* in infected mouse liver

gene	category	function	6 h.p.i.		24 h.p.i.		48 h.p.i.	
			Fold	FDR p-val.	Fold	FDR p-val.	Fold	FDR p-val.
Trf	Metal sequestration	transferrin	1.4	1.000	-1.0	1.000	3.6	0.017
Ltf		lactotransferrin	23.7	0.029	32.7	0.002	407.1	<0.001
Lcn2		lipocalin 2	162.2	<0.001	349.7	<0.001	861.9	<0.001
Hamp		hepcidin antimicrobial peptide	-1.6	0.802	-2.1	0.713	1.3	1.000
Tlr2	Innate immunity	toll-like receptor 2	140	<0.001	8.4	<0.001	8.0	<0.001
Myd88		myeloid differentiation primary response gene 88	7.1	<0.001	2.3	0.037	3.6	0.001
Saa3		serum amyloid A 3	370	<0.001	840	<0.001	3300	<0.001
Nos2		nitric oxide synthase 2, inducible	234	<0.001	200	<0.001	610	<0.001
mt-Cytb	Respiratory electron transport	mitochondrially encoded cytochrome b	-17	<0.001	-10	<0.001	-21	<0.001
Uqcrq		ubiquinol-cytochrome c reductase, complex III subunit VII	-1.8	0.661	-3.9	0.001	-16	<0.001
mt-ND4l		mitochondrially encoded NADH dehydrogenase 4L	-15	<0.001	-5.5	<0.001	-18	<0.001
Hilpda	Hypoxia	hypoxia inducible lipid droplet associated	2.3	0.440	5.3	0.002	11.7	<0.001
Hif1a		hypoxia inducible factor 1, alpha subunit	1.3	1.000	1.4	0.835	4.1	<0.001
Hif3a		hypoxia inducible factor 3, alpha subunit	-4.1	0.175	8.1	0.001	4.0	0.157

Supplementary Table 2| Gene expression change in representative genes in *S. aureus*-infected mice compared with PBS-injected mice.

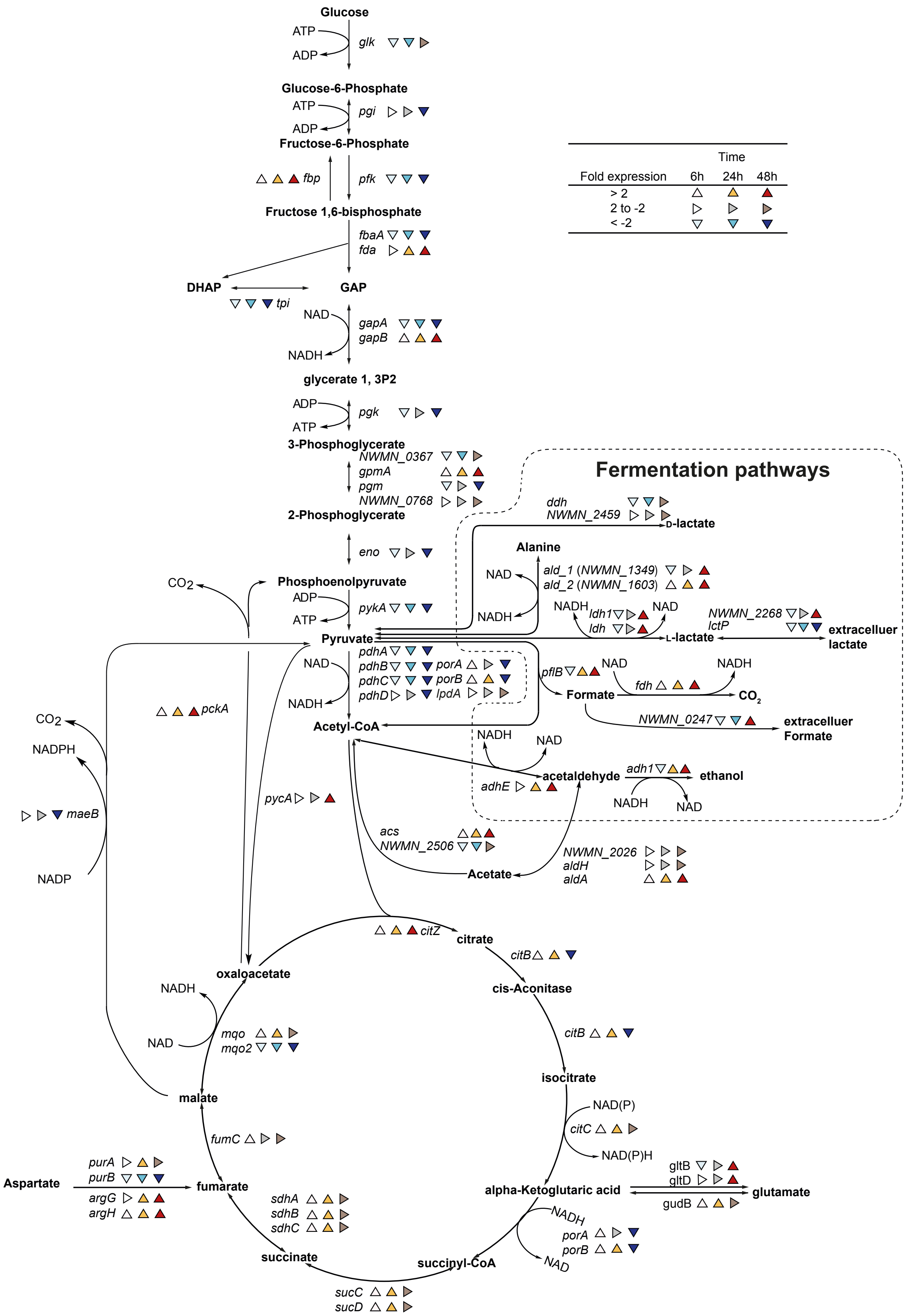

Supplementary Figure 1| Gene expression changes related to glycolysis and the TCA cycle in infected mouse liver compared with culture medium

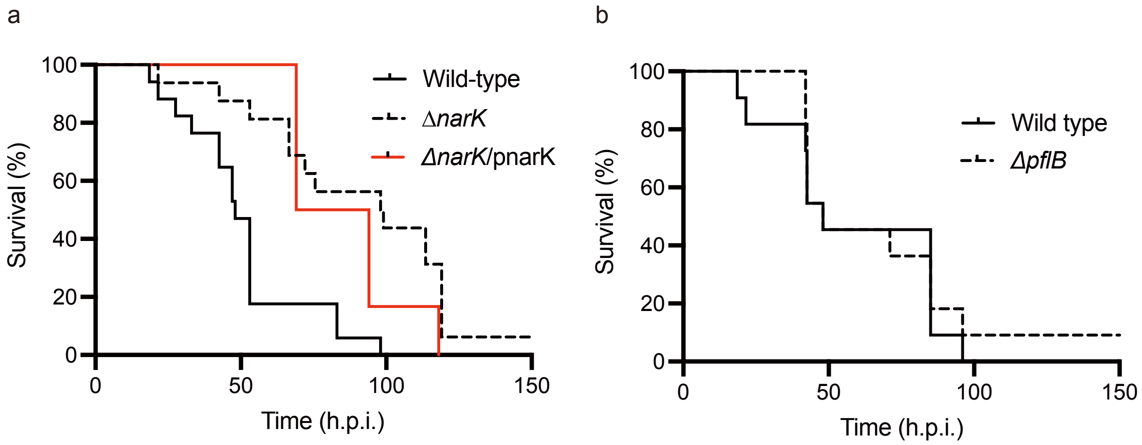

Supplementary Figure 2| Mouse-killing ability of a disruption mutant of the *narK* and *pflB* gene involved in anaerobic metabolism

a, Survival curves of mice injected with wild-type, *narK* gene-disrupted strains, and the Δ*narK*/*narK* complement strain via tail vein. For the wild-type and Δ*narK* strains, the results of 3 independent experiments were combined (wild-type: 6.0x10^7, 4.3x10^7 and 3.7x10^7 n=17, Δ*narK*: 6.0x10^7, 4.6x10^7 and 3.9x10^7 n=16, respectively) and for the Δ*narK*/pnarK strain, a single experiment was performed (4.8x10^7 CFU n=6). Statistical analysis was performed by the log-rank test (*p*=0.0003 chi square=13.72, df=1 between WT and Δ*narK* strain, and p=0.2635 chi square=1.250, df=1 between the Δ*narK*/pnarK and Δ*nark* strains). **b**, Parent and Δ*plfB* strains were intravenously injected. The combined results of 2 independent experiments are shown (n=11). Injection doses were 3.8x10^7 and 4.7x10^7CFU for the wild-type, and 3.2x10^7 and 4.5x10^7CFU for the mutant. No significant difference was detected by log-rank test (p=0.6644, chi square=0.1882, df=1).

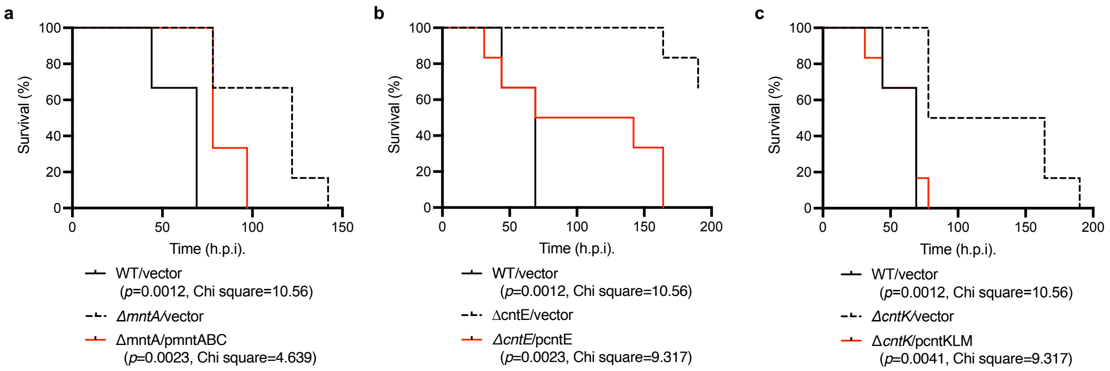

Supplementary Figure 3| Recovery of virulence by gene complementation

Comparison of mouse-killing ability between gene-disrupted mutants, Δ*mntA* (a), Δ*cntE* (b), and Δ*cntK* (c) and their gene-complemented strains. Bacterial suspensions were intravenously injected. Injection doses were $5.6x10^7$ CFU for the wild-type (WT)/vector strain, $5.9x10^7$ CFU for the Δ*mntA*/ vector, $5.9x10^7$ CFU for Δ*mntA*/pmntABC, 6.1 $x10^7$ CFU for the Δ*cntE*/vector, $5.7x10^7$ CFU for Δ*cntE*/pcntE, $5.6x10^7$ CFU for the Δ*cntK*/vector, and $4.4x10^7$ CFU for Δ*cntK*/pcntKLM. The displayed statistical analyses were performed by the Log-rank (Mantel-Cox) test against disruptant mutant with vector (n=6 in each group and all df=1).

Gene disruption or plasmid construction	Primer name	Sequence (5'->3')
pflB	0162_F	GTGTCCCTTAAGCATAGT
	0162_R	CAGATGGAGGCGTTTAGT
mntA	0603_F	CTACAGTCAGTGCTACTC
	0603_R	TGGTGCTGGTAAATCTTC
narK	2288_F	TTTTTGTCGACCTACGTTCTTGTGTGTCACC
	2288_R	TTTTTGGATCCGTTTAGTGGTTGGGTTTATGG
cntE	2359_F	TTTTTGTCGACCGCGATAGCCAAAGAATCT
	2359_R	TTTTTGGATCCCTTGGCCCTTTTGAGAT
cntK	2367_F	ACTAAATACTGCCCCCT
	2367_R	ATGCATCTATCGCCAATC
pmntABC	0603_Fw_Hiind	ATATAAGCTTTTATGTTGATGTGTGGCCT
	0603_Rev_BamH	AATTGGATCCACGTCCTACCATTTCACT
pnarK	narK_Fw_Eco	CCGGAATTCCCTCCTTATGTTGTCAGC
	narK_Rev_Hind	CGCAAGCTTCCATTACCTTGCCTAAAATC
pcntE	cntE_Fw_Eco	CGCGGATCCATGAAAGGTGCAATGGCTTGGCC
	cntE_Rev_salI	CCGGTCGACTTAAAGACTACTCGCTGGACGTGG
pcntLMK	cntLMK_Fw_Hind	ATATAAGCTTCAACCAGTTAAAATTAGCCC
	cntLMK_Rev_BamH	AATTGGATCCGTAGAGCCACAAGAAGCAA

Supplementary Table 3| Primers used in this study

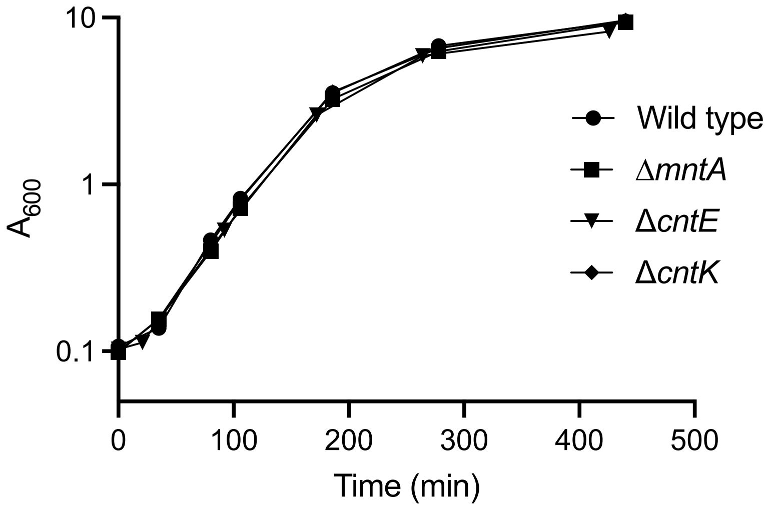

Supplementary Figure 4| Growth curve of gene-disruptant mutants used in this study

Full growth cultured cells were diluted 100-times in TSB medium and absorbance at 600 nm was monitored by a photometer. Representative data of 2 independent experiments with similar results are shown.
