# Supplementary material for: Transcriptome change of Staphylococcus aureus in infected mouse liver
Source: Commun Biol. 2022 Jul 20;5:721. doi: 10.1038/s42003-022-03674-5 (PMC9300722; doi:10.1038/s42003-022-03674-5)
Supplement: Supplementary file 3 — Description of Additional Supplementary Files [file 42003_2022_3674_MOESM3_ESM.pdf]

Description of Additional Supplementary Files

File name: Supplementary Data 1
Description: in vivo RNA-Seq results of S. aureus in infected mouse liver.

File name: Supplementary Data 2
Description: Gene expression change in liver of mice after S. aureus infection compared with PBS treated mice.

File name: Supplementary Data 3
Description: Reactome analysis of genes upregulated or downregulated after S. aureus infection.

File name: Supplementary Data 4
Description: The source data behind the graphs in the paper.
